# Adenosine A_2A_ Receptor Deletion Blocks the Beneficial Effects of *Lactobacillus reuteri* in Regulatory T-Deficient Scurfy Mice

**DOI:** 10.3389/fimmu.2017.01680

**Published:** 2017-12-06

**Authors:** Baokun He, Thomas K. Hoang, Dat Q. Tran, Jon Marc Rhoads, Yuying Liu

**Affiliations:** ^1^Division of Gastroenterology, Department of Pediatrics, McGovern Medical School, The University of Texas Health Science Center at Houston, Houston, TX, United States

**Keywords:** regulatory T deficiency, autoimmunity, adenosine A_2A_ receptor, *Lactobacillus reuteri*, cytokines, IPEX, scurfy, probiotic

## Abstract

The lack of a functional Foxp3 transcription factor and regulatory T (Treg) cells causes lethal, CD4^+^ T cell-driven autoimmune diseases in scurfy (SF) mice and humans. Recent studies have shown that adenosine A_2A_ receptor activation limits inflammation and tissue damage, thereby playing an anti-inflammatory role. However, the role of the adenosine A_2A_ receptor in the development of disease in SF mice remains unclear. Using a genetic approach, we found that adenosine A_2A_ receptor deletion in SF mice (SF$\cdot {\text{A}}_{{\text{2A}}^{\text{-/-}}}$) does not affect early life events, the development of a lymphoproliferative disorder, or hyper-production of pro-inflammatory cytokines seen in the Treg-deficiency state. As shown previously, *Lactobacillus reuteri* DSM 17938 treatment prolonged survival and reduced multiorgan inflammation in SF mice. In marked contrast, A_2A_ receptor deletion completely blocked these beneficial effects of *L. reuteri* in SF mice. Altogether, these results suggest that although absence of the adenosine A_2A_ receptor does not affect the development of disease in SF mice, it plays a critical role in the immunomodulation by *L. reuteri* in Treg-deficiency disease. The adenosine A_2A_ receptor and its activation may have a role in treating other Treg dysfunction-mediated autoimmune diseases.

## Introduction

Foxp3^+^ regulatory T (Treg) cells play a pivotal role in the phenomenon of self-tolerance. In humans, Foxp3 mutations result in immunodysregulation, polyendocrinopathy, and enteropathy, with X-linked inheritance (called IPEX syndrome). Newborn boys with IPEX syndrome have severe enteropathy, eczema, type I diabetes, thyroiditis, hemolytic anemia, and thrombocytopenia; and they die within the first years of life if left untreated (1, 2). In the mouse model, Foxp3-deficient scurfy (SF) mice develop a lethal autoimmune disease which closely resembles the IPEX syndrome (3, 4). SF mice develop early-onset dermatitis, progressive multiorgan inflammation, and early death within the first month of life due to a lymphoproliferative syndrome. This lethal lymphoproliferative syndrome is predominately mediated by CD4^+^ T cells in humans and mice (5, 6). Consequently, the SF mouse is a valuable model for studying novel therapies for human IPEX syndrome and other autoimmune diseases associated with Treg deficiency. These include IPEX-like syndromes induced by mutations or deficiency in Itchy E3 ubiquitin protein ligase (ITCH), the α-chain of the IL-2 receptor (CD25), signal transducer and activator of transcription 5b, STAT1, or cytotoxic T-lymphocyte-associated protein 4 (7, 8).

High levels of the adenosine A_2A_ receptor are found in the brain, thymus, and spleen, as well as in circulating platelets and leukocytes (9). On the cell membrane of murine T lymphocytes, the adenosine A_2A_ receptor is highly expressed and is increased by T-cell receptor (TCR) stimulation (10, 11). In humans, the A_2A_ receptor is more highly expressed in CD4^+^ compared to CD8^+^ T cells (12). Moreover, numerous studies have highlighted the anti-inflammatory role of the adenosine A_2A_ receptor (13, 14). There have been observations of anti-inflammatory effects of A_2A_ receptor agonists *in vivo* and, conversely, enhanced inflammation in A_2A_ receptor knockout mice (14). However, the function of adenosine A_2A_ receptor in the development and control of autoimmune diseases remains unclear.

Recently, probiotics have emerged as relatively safe and inexpensive treatments for a number of gastrointestinal conditions. *Lactobacillus reuteri* strain DSM 17938 (*L. reuteri*) is a probiotic originally isolated from a Peruvian mother’s breast milk (15). This probiotic has been shown to prevent necrotizing enterocolitis (NEC) in newborn animals (16, 17) by inhibiting the toll-like receptor 4-mediated NF-κB pathway, facilitating the induction of immune-modulating Foxp3^+^ Tregs, and lowering the number of pro-inflammatory effector-memory T-cells in the intestinal mucosa. In humans, *L. reuteri* has been shown to reduce the severity of acute infant diarrhea (18–20), to prevent NEC in premature infants (21–23), and to decrease crying time in infants with colic (24, 25).

In addition, our recent studies demonstrated that *L. reuteri* significantly prolongs the survival rate of the SF mouse (from less than 30 days to greater than 4 months of age) by suppression of inflammatory T cells (mainly T_H_1 and T_H_2) extensively activated in multiple organs of SF mice (7). Mechanistically, *L. reuteri* modulates the abnormal microbial communities associated with these diseases, stimulating the production of bioactive metabolites involved in immune modulation. We observed that inosine, a downstream metabolite of adenosine, was decreased in the plasma of SF mice compared to wild-type (WT) mice, but was increased by oral administration of *L. reuteri* to SF mice. Oral administration of inosine by itself prolonged the survival and decreased autoimmunity of SF mice. Inosine was found to be a critical effector molecule of *L. reuteri* treatment, altering T_H_1/T_H_2 cell differentiation by activating A_2A_ receptors, predominately expressed on T cells. Blocking A_2A_ receptors by an A_2A_ antagonist reversed the anti-inflammatory effects of both inosine and *L. reuteri*, indicating that A_2A_ receptor appears to play a critical role in the beneficial effects of *L. reuteri* in the SF model (7).

In this study, we produced SF mice with genetically deleted adenosine A_2A_ receptor (SF$\cdot {\text{A}}_{{\text{2A}}^{\text{-/-}}}$) to conclusively provide evidence of a central role of A_2A_ receptor in the actions of *L. reuteri*. We demonstrate that A_2A_ receptor gene deletion in SF mice did not accentuate the development of disease, but prevented the inhibitory effects of *L. reuteri* on autoimmunity. Our study highlights the A_2A_ receptor as a key mediator of the immunomodulatory mechanism of this probiotic.

## Materials and Methods

### Animals

Wild-type C57BL/6, heterozygous B6.Cg-Foxp3^sf^/J and adora2a^tm1Jfc^/J mice were purchased from Jackson Laboratories and allowed to acclimatize for 2 weeks before experimentation. SF mice were bred with adora2a^tm1Jfc^/J mice to generate adenosine A_2A_ receptor-deficient SF mice (${\text{A}}_{{\text{2A}}^{\text{-/-}}}$ SF mice, SF$\cdot {\text{A}}_{{\text{2A}}^{\text{-/-}}}$). All males were either SF/SF$\cdot {\text{A}}_{{\text{2A}}^{\text{-/-}}}$ double knockouts, the experimental group, or WT/${\text{A}}_{{\text{2A}}^{\text{-/-}}}$ littermates, used as controls. All mice were housed in the animal facility at UT Health Science Center at Houston. This study was carried out in accordance with the recommendations of the Guide for the Care and Use of Laboratory Animals (NIH) and The Institutional Animal Care and Use Committee (IACUC). The protocol was approved by the IACUC (protocol numbers: AWC-14-056 and AWC-17-0045).

### *L. reuteri* Treatment of SF Mice

*Lactobacillus reuteri* DSM17938 (*L. reuteri*), originally isolated from human breast milk, was provided by BioGaia AB (Stockholm, Sweden) and prepared as described previously (7). Each mouse was given either De Man, Rogosa, and Sharpe agar (MRS) media as a control or *L. reuteri* (SF + LR or SF$\cdot {\text{A}}_{{\text{2A}}^{\text{-/-}}}$ + LR) which was given by daily gavage in cultured media (10^7^ CFU/day), starting from 8 to 20 days of age for tissue analysis or to infinity for survival.

### Histopathology

All tissues of WT, SF, SF + LR, ${\text{A}}_{{\text{2A}}^{\text{-/-}}}$, SF$\cdot {\text{A}}_{{\text{2A}}^{\text{-/-}}}$, and SF$\cdot {\text{A}}_{{\text{2A}}^{\text{-/-}}}$ + LR mice were fixed and stained with hematoxylin and eosin (H&E) for histological evaluation by the Cellular and Molecular Morphology Core Lab (The Texas Medical Center Digestive Diseases Center, Houston, TX, USA). The area of lymphocyte infiltration in liver and lung was assessed in a blinded fashion using Image J morphometry software (NIH, USA).

### *In vitro* Tissue Preparation and Stimulation for Flow Cytometry Analysis

Single-cell suspensions from the spleen were prepared by gently fragmenting and filtering the tissues through 40-μm cell strainers (BD Bioscience) into MACS buffer (1× PBS, 0.5% bovine BSA, and 2 mM EDTA). For *in vitro* stimulation of splenocytes, cells were stimulated with 50 ng/mL of phorbol 12-myristate 13-acetate (PMA) and 1 μg/mL of ionomycin in the presence of brefeldin A (5 μ/mL) for 4 h to analyze IFN-γ-producing (T_H_1) and IL-4-producing (T_H_2) CD4^+^ T cells by flow cytometry.

### Staining Cells for Flow Cytometry Analysis

For evaluation of T_H_1 and T_H_2 cells, cells were surface stained by fluorescein-labeled CD4. Intracellular staining was performed with a fixation/permeabilization kit, according to the manufacturer’s protocol (eBioscience) and stained with IFN-γ and IL-4 for T_H_1 and T_H_2 cells, respectively. The data from all samples were acquired on BD FACSCalibur and analyzed using FlowJo software (TreeStar, Inc.).

### Plasma Cytokine Assays

Plasma cytokine levels of IFN-γ, IL-1β, IL-2, IL-4, IL-5, IL-10, and IL-12p70 were assessed using a mouse multi-spot pro-inflammatory panel kit, and signals were detected by Imager 2400 from Meso Scale Discovery, according to the manufacturer’s protocol.

### Statistical Analysis

Data are presented as mean ± SEM. Statistical significance was determined using one-way ANOVA corrected for multiple comparisons with Tukey and Dunnett’s posttests. The statistical analysis was performed using Prism version 4.0 (GraphPad Software). A *p*-value <0.05 was considered to indicate statistical significance.

## Results

### Adenosine A_2A_ Receptor Deletion Does Not Affect Early Death in SF Mice

**Figure 1:**
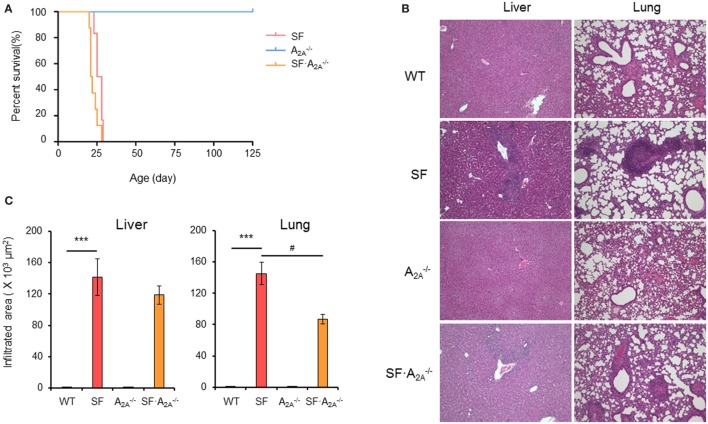
Effect of adenosine A_2A_ receptor deletion on the development of diseases in scurfy (SF) mice. **(A)** Survival curves of ${\text{A}}_{{\text{2A}}^{\text{-/-}}}$, SF, and SF$\cdot {\text{A}}_{{\text{2A}}^{\text{-/-}}}$ mice (*n* = 6–9). **(B)** H&E staining of representative sections of liver and lung of wild-type (WT), SF, ${\text{A}}_{{\text{2A}}^{\text{-/-}}}$, and SF$\cdot {\text{A}}_{{\text{2A}}^{\text{-/-}}}$ mice (*n* = 6–9). **(C)** Quantitation of inflammatory infiltrates in liver and lung of WT, SF, ${\text{A}}_{{\text{2A}}^{\text{-/-}}}$, and SF$\cdot {\text{A}}_{{\text{2A}}^{\text{-/-}}}$ mice (*n* = 6–9). Data are presented as mean ± SEM. ***p < 0.001. SF vs. WT. ^#^*p* < 0.05. SF$\cdot {\text{A}}_{{\text{2A}}^{\text{-/-}}}$ vs. SF.

To determine the role of the adenosine A_2A_ receptor in the pathogenesis of autoimmunity in the SF mouse, we bred female (Foxp3^sf/+^) mice with adora2a gene knockout $\left({\text{A}}_{{\text{2A}}^{\text{-/-}}}\right)$ mice. The male adenosine A_2A_ receptor-deficient SF (SF$\cdot {\text{A}}_{{\text{2A}}^{\text{-/-}}}$) mice died between 21 and 25 days of age (Figure 1A). Our data show that A_2A_ receptor deletion does not enhance or reverse the effect of the lethal autoimmune disease as it relates to lifespan in the SF mouse.

### Adenosine A_2A_ Receptor Deletion Regulates Organ-Specific Inflammation in SF Mice

Scurfy mice develop severe inflammation in several tissues, including liver, lung, ear, tail, intestine, and colon (26). To examine whether adenosine A_2A_ receptor deletion alters the autoimmune damage in these tissues, we measured the area of inflammatory cell infiltration in H&E-stained tissues sections from WT, SF, ${\text{A}}_{{\text{2A}}^{\text{-/-}}}$, and SF$\cdot {\text{A}}_{{\text{2A}}^{\text{-/-}}}$ mice at the 20 days of age. There were no inflammatory infiltrates in the liver, lung, ear, tail, and intestine in ${\text{A}}_{{\text{2A}}^{\text{-/-}}}$ mice (Figures 1B,C; Figure S1 in Supplementary Material). Indeed, the area of inflammatory cell infiltration in most organs studied (liver, ear, tail, and intestine) in SF$\cdot {\text{A}}_{{\text{2A}}^{\text{-/-}}}$ mice was similar to the inflammatory cell infiltrate in SF mice (Figures 1B,C; Figure S1 in Supplementary Material). However, the inflammatory cell infiltration of the lung was slightly reduced in SF$\cdot {\text{A}}_{{\text{2A}}^{\text{-/-}}}$ mice compared to SF mice. These results demonstrate that the A_2A_ receptor deletion does not have a major impact on inflammation in SF mice.

### Adenosine A_2A_ Receptor Deletion Does Not Reduce T_H_1/T_H_2 Cells in SF Mice

**Figure 2:**
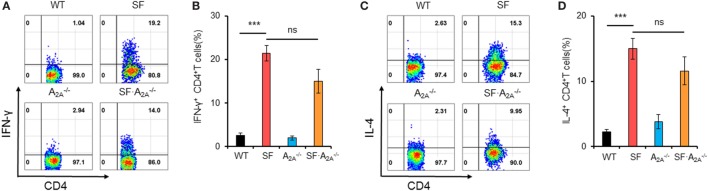
Effect of adenosine A_2A_ receptor deletion on T_H_1/T_H_2 cells in spleen of scurfy (SF) mice. **(A)** Representative FACS plots of IFN-γ-producing CD4^+^ T (T_H_1) cells in spleen of wild-type (WT), SF, ${\text{A}}_{{\text{2A}}^{\text{-/-}}}$, and SF$\cdot {\text{A}}_{{\text{2A}}^{\text{-/-}}}$ mice. **(B)** Percentage of T_H_1 cells in spleen of WT, SF, ${\text{A}}_{{\text{2A}}^{\text{-/-}}}$, and SF$\cdot {\text{A}}_{{\text{2A}}^{\text{-/-}}}$ mice (*n* = 6–9). **(C)** Representative FACS plots of IL-4-producing CD4^+^ T (T_H_2) cells in spleen of WT, SF, ${\text{A}}_{{\text{2A}}^{\text{-/-}}}$, and SF$\cdot {\text{A}}_{{\text{2A}}^{\text{-/-}}}$ mice. **(D)** Percentage of T_H_2 cells in spleen of WT, SF, ${\text{A}}_{{\text{2A}}^{\text{-/-}}}$, and SF$\cdot {\text{A}}_{{\text{2A}}^{\text{-/-}}}$ mice (*n* = 6–9). Data are presented as mean ± SEM. ***p < 0.001. SF vs. WT. ns, non-significance.

The lethal lymphoproliferative syndrome in SF mice is predominantly caused by CD4^+^ T cell-induced pathology (27, 28). To evaluate the effect of A_2A_ receptor deletion on T_H_1/T_H_2 cells in SF mice, we measured the percentage of IFN-γ-producing CD4^+^ T (T_H_1) cells and IL-4-producing CD4^+^ T (T_H_2) cells in the spleen of WT, SF, ${\text{A}}_{{\text{2A}}^{\text{-/-}}}$, and SF$\cdot {\text{A}}_{{\text{2A}}^{\text{-/-}}}$ mice. A_2A_ receptor deletion did not change the frequency of T_H_1 or T_H_2 cells in WT or SF mice (WT$\cdot {\text{A}}_{{\text{2A}}^{\text{-/-}}}$ or SF$\cdot {\text{A}}_{{\text{2A}}^{\text{-/-}}}$) mice, respectively (Figure 2). Our findings suggest that A_2A_ receptor deletion does not inhibit T_H_1/T_H_2 cell proliferation in SF mice.

### Adenosine A_2A_ Receptor Deletion Alters the Majority of Pro-inflammatory Cytokines in SF Mice

**Figure 3:**
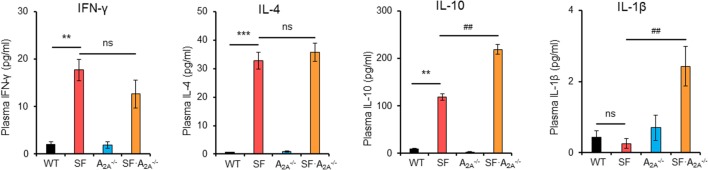
Effect of adenosine A_2A_ receptor deletion on pro-inflammatory cytokines in scurfy (SF) mice. Plasma levels of IFN-γ, IL-1β, IL-4, and IL-10 in wild-type (WT), SF, ${\text{A}}_{{\text{2A}}^{\text{-/-}}}$, and SF$\cdot {\text{A}}_{{\text{2A}}^{\text{-/-}}}$ mice were quantified by a mouse multi-spot pro-inflammatory panel kit (*n* = 6–9). Data are presented as mean ± SEM. **p < 0.01, ***p < 0.001. SF vs. WT. ^#^*p* < 0.05, ^##^*p* < 0.01. SF$\cdot {\text{A}}_{{\text{2A}}^{\text{-/-}}}$ vs. SF. ns, non-significance.

After TCR stimulation, CD4^+^ T cells from SF mice produce high levels of cytokines, including IFN-γ, IL-2, IL-4, IL-10, and TNF-α (29, 30). To examine whether these pro-inflammatory cytokines reached higher levels in SF$\cdot {\text{A}}_{{\text{2A}}^{\text{-/-}}}$ mice compared to SF mice, we measured the concentration of pro-inflammatory cytokines in plasma (Figure 3; Figure S2 in Supplementary Material). Our results demonstrated that the levels of IFN-γ, IL-2, IL-4, IL-5, and IL-10 were increased in SF mice compared to WT mice. Conversely, the levels of IL-1β and IL-12p70 were not increased in SF mice compared to WT mice. However, A_2A_ receptor deletion increased the levels of pro-inflammatory IL-1β and anti-inflammatory cytokine IL-10 in SF$\cdot {\text{A}}_{{\text{2A}}^{\text{-/-}}}$ mice compared to SF mice. Together, our results show that the majority of pro-inflammatory cytokines contribute to the development of disease in SF mice.

### Adenosine A_2A_ Receptor Deletion Reverses the Effect of *L. reuteri* on Lifespan in SF Mice

**Figure 4:**
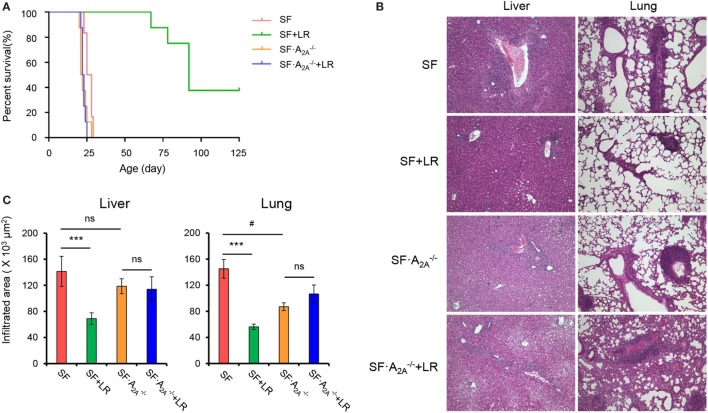
Adenosine A_2A_ receptor deletion blocks effects of *Lactobacillus reuteri* on scurfy (SF) mice. **(A)** Survival curves of SF, SF + LR, SF$\cdot {\text{A}}_{{\text{2A}}^{\text{-/-}}}$, and SF$\cdot {\text{A}}_{{\text{2A}}^{\text{-/-}}}$ + LR mice (*n* = 6–9). **(B)** H&E staining of representative sections of liver and lung of SF, SF + LR, SF$\cdot {\text{A}}_{{\text{2A}}^{\text{-/-}}}$, and SF$\cdot {\text{A}}_{{\text{2A}}^{\text{-/-}}}$ + LR mice (*n* = 6–9). **(C)** Quantitation of inflammatory infiltrates in liver and lung of SF, SF + LR, SF$\cdot {\text{A}}_{{\text{2A}}^{\text{-/-}}}$, and SF$\cdot {\text{A}}_{{\text{2A}}^{\text{-/-}}}$ + LR mice (*n* = 6–9). Data are presented as mean ± SEM. ***p < 0.001. SF + LR vs. SF. ns, non-significance. ^#^*p* < 0.05.

Previous studies have suggested that *L. reuteri* increases survival in SF mice by restoring plasma levels of the nucleotide inosine, which is an adenosine A_2A_ receptor agonist (7), which represents a novel mechanism of action of probiotics. However, the effect of targeted genetic deletion of A_2A_ receptor on the beneficial effects of *L. reuteri* in SF mice is unknown. To examine this effect, we fed SF with *L. reuteri* (SF + LR) and SF$\cdot {\text{A}}_{{\text{2A}}^{\text{-/-}}}$ mice with *L. reuteri* (SF$\cdot {\text{A}}_{{\text{2A}}^{\text{-/-}}}$ + LR). The median lifespan of the SF mouse was significantly increased by LR feeding (SF + LR mice), from 26.5 to 92 days (*p* < 0.001). However, the median lifespan of SF$\cdot {\text{A}}_{{\text{2A}}^{\text{-/-}}}$ mice with *L. reuteri* treatment (SF$\cdot {\text{A}}_{{\text{2A}}^{\text{-/-}}}$ + LR) was 22.5 days (Figure 4A). These data demonstrate that A_2A_ receptor plays a critical role in the effect of *L. reuteri* to prolong the lifespan of the SF mouse.

### Adenosine A_2A_ Receptor Deletion Negates the Effect of *L. reuteri* on Inflammation in SF Mice

We next asked whether A_2A_ receptor deletion could inhibit the beneficial effect of *L. reuteri* on multiorgan inflammation in living SF mice. Therefore, we fed SF and SF$\cdot {\text{A}}_{{\text{2A}}^{\text{-/-}}}$ mice with a daily dose of *L. reuteri*, starting from 8 to 20 days. H&E-stained tissue sections from SF, SF + LR, SF$\cdot {\text{A}}_{{\text{2A}}^{\text{-/-}}}$, and SF$\cdot {\text{A}}_{{\text{2A}}^{\text{-/-}}}$ + LR groups were scored. Inflammatory cell infiltration of liver and lung was reduced in SF + LR mice compared to SF mice. However, this infiltration was not reduced in SF$\cdot {\text{A}}_{{\text{2A}}^{\text{-/-}}}$ + LR mice compared to SF$\cdot {\text{A}}_{{\text{2A}}^{\text{-/-}}}$ mice (Figures 4B,C). These results demonstrate that A_2A_ receptor activation contributes to the inhibition by *L. reuteri* of inflammation in the SF mouse.

### Adenosine A_2A_ Receptor Deletion Inhibits *L. reuteri*-Mediated Reduction of T_H_1/T_H_2 Splenocytes in SF Mice

**Figure 5:**
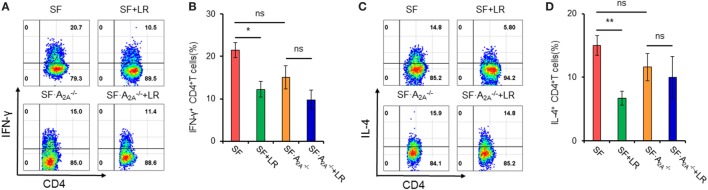
Effect of *Lactobacillus reuteri* on T_H_1/T_H_2 cells in spleen of scurfy (SF) and SF$\cdot {\text{A}}_{{\text{2A}}^{\text{-/-}}}$ mice. **(A)** Representative FACS plots of IFN-γ-producing CD4^+^ T (T_H_1) cells in spleen of SF, SF + LR, SF$\cdot {\text{A}}_{{\text{2A}}^{\text{-/-}}}$, and SF$\cdot {\text{A}}_{{\text{2A}}^{\text{-/-}}}$ + LR mice. **(B)** Percentage of T_H_1 cells in spleen of SF, SF + LR, SF$\cdot {\text{A}}_{{\text{2A}}^{\text{-/-}}}$, and SF$\cdot {\text{A}}_{{\text{2A}}^{\text{-/-}}}$ + LR mice (*n* = 6–9). **(C)** Representative FACS plots of IL-4-producing CD4^+^ T (T_H_2) cells in spleen of SF, SF + LR, SF$\cdot {\text{A}}_{{\text{2A}}^{\text{-/-}}}$, and SF$\cdot {\text{A}}_{{\text{2A}}^{\text{-/-}}}$ + LR mice. **(D)** Percentage of T_H_2 cells in spleen of SF, SF + LR, SF$\cdot {\text{A}}_{{\text{2A}}^{\text{-/-}}}$, and SF$\cdot {\text{A}}_{{\text{2A}}^{\text{-/-}}}$ + LR mice (*n* = 6–9). Data are presented as mean ± SEM. *p < 0.05, **p < 0.01. SF + LR vs. SF. ns, non-significance.

Our studies have shown that *L. reuteri* reduces T_H_1/T_H_2 cells in SF mice (7). To explore whether genetic deletion of A_2A_ receptor contributes to the inhibition of *L. reuteri* of T_H_1/T_H_2 cell differentiation in SF mice, we measured the frequency of T_H_1/T_H_2 cells in the spleen from SF, SF + LR, SF$\cdot {\text{A}}_{{\text{2A}}^{\text{-/-}}}$, and SF$\cdot {\text{A}}_{{\text{2A}}^{\text{-/-}}}$ + LR mice (Figure 5). *L. reuteri*-treated SF mice had reduced T_H_1/T_H_2 cells when compared to SF mice at 20 days of age, consistent with our previous studies (7). Interestingly, *L. reuteri* treatment did not reduce the percentage of T_H_1/T_H_2 cells in SF$\cdot {\text{A}}_{{\text{2A}}^{\text{-/-}}}$ mice, demonstrating that the activated A_2A_ receptor plays an important role in *L. reuteri*-mediated immunoregulation in SF mice.

### Adenosine A_2A_ Receptor Deletion Reverses the Effect of *L. reuteri* on Pro-inflammatory Cytokines in SF Mice

**Figure 6:**
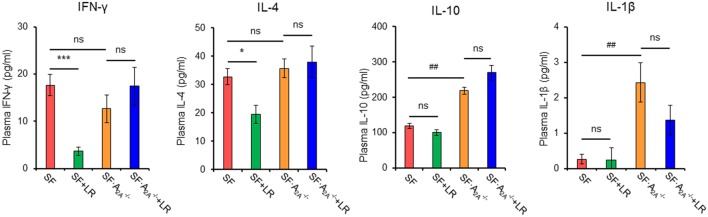
Effect of *Lactobacillus reuteri* on pro-inflammatory cytokines in scurfy (SF) and SF$\cdot {\text{A}}_{{\text{2A}}^{\text{-/-}}}$ mice. Plasma levels of IFN-γ, IL-4, IL-1β, and IL-10 in SF, SF + LR, SF$\cdot {\text{A}}_{{\text{2A}}^{\text{-/-}}}$, and SF$\cdot {\text{A}}_{{\text{2A}}^{\text{-/-}}}$ + LR mice were quantified by a mouse multi-spot pro-inflammatory panel kit (*n* = 6–9). Data are presented as mean ± SEM. *p < 0.05, ***p < 0.001. SF + LR vs. SF. ^##^*p* < 0.01. SF$\cdot {\text{A}}_{{\text{2A}}^{\text{-/-}}}$ vs. SF. ns, non-significance.

To test whether cytokine production regulated by *L. reuteri* treatment depends on the A_2A_ receptor in SF mice, we examined plasma cytokines from SF, SF + LR, SF$\cdot {\text{A}}_{{\text{2A}}^{\text{-/-}}}$, and SF$\cdot {\text{A}}_{{\text{2A}}^{\text{-/-}}}$ + LR mice (Figure 6; Figure S3 in Supplementary Material). *L. reuteri*-treated SF mice had reduced levels of IFN-γ and IL-4 and increased the levels of IL-12p70, but they had no changes in the levels of IL-1β, IL-2, IL-5, and IL-10, when compared to SF mice. Notably, A_2A_ receptor deletion reversed the effects of *L. reuteri* on IFN-γ, IL-4, and IL-12p70. These findings further substantiate that A_2A_ receptor activation contributes to the inhibitory effects of *L. reuteri* on inflammation in the SF mouse.

## Discussion

This study demonstrated a central role of the adenosine A_2A_ receptor in mediating the protection of probiotic *L. reuteri* against inflammation in the Treg-deficient SF mouse (a model of human IPEX syndrome), evidenced by the observation that SF mice with an A_2A_ receptor deletion continued to have systemic inflammation which was unresponsive to *L. reuteri* treatment.

It is well known that the lethal lymphoproliferative syndrome characterizing SF mice is predominately mediated by T_H_1 and T_H_2 cell-induced pathology (27, 28). The key to Treg suppression of T effector cells (T_H_1/T_H_2/T_H_17) is an interaction between adenosine produced by Tregs (mediated by a CD39–CD73 pathway) and the A_2A_ receptor expressed on nearby T effector cells (31). Lymphocytes predominately express A_2A_ receptors (10–12, 32, 33). However, during Treg deficiency in SF mice or human IPEX syndrome, T_H_1 and T_H_2 cells lose their regulation by adenosine A_2A_-mediated signaling, resulting in T_H_1 and T_H_2 cell-induced pathology. Studies by Csoka et al. showed that an agonist of A_2A_ receptors inhibited the proliferation and effector functions of CD4^+^ T cells isolated from WT mice but failed to block these of cells obtained from A_2A_ knockout mice (33), indicating that the activated adenosine A_2A_ receptor plays a critical role in the suppression of T_H_1 and T_H_2 cells.

Our previous study demonstrated that Treg deficiency induces gut microbial dysbiosis dynamically over the first 22 days of life, an effect which could be reprogrammed by oral administration of *L. reuteri*. *L. reuteri* suppressed T_H_1 and T_H_2 cells in SF mice, as evidenced by lower circulating levels of IFN-γ (T_H_1) and IL-4 (T_H_2) and reduced numbers of IFN-γ and IL-4-expressing lymphocytes in spleen and mesenteric lymph nodes of SF mice. Metabolites produced by *L. reuteri* or *L. reuteri*-modulated bacteria are known to promote or suppress immune cell function (34–36). We discovered that the purine metabolite inosine, a metabolite of adenosine, is severely decreased in SF mice, while increased after oral administration of *L. reuteri* (7). Inosine has been proved to be a functional agonist of the A_2A_ receptor which has an anti-inflammatory effect (37–43). Our previous experiments by using adenosine receptor knockout mice to study the suppression of inosine on naïve CD4^+^ T cell differentiation into T_H_1 and T_H_2 *in vitro* strongly suggested that the effects of inosine are dependent on the A_2A_ receptor on T cells (7). In addition, an *in vivo* study showed that an A_2A_ receptor antagonist blocks the anti-inflammatory effects of both inosine and (*L. reuteri* DSM 17938) on T_H_1 and T_H_2 suppression and multiorgan lymphocyte infiltration in SF mice (7). In summary, the A_2A_ receptor mediates the beneficial biological effects of *L. reuteri* and inosine in SF mice. In this study, we further confirmed a critical role of A_2A_ receptor-mediated effects by genetic deletion of A_2A_ in SF mice (SF$\cdot {\text{A}}_{{\text{2A}}^{\text{-/-}}}$ mice).

Mechanistically, how *L. reuteri* results in increased serum level of inosine is not fully understood. When we compared *L. reuteri* cultures to MRS broth (culture media without *L. reuteri*) after 16 h of anaerobic growth, *L. reuteri* did not generate significant amounts of purines or inosine in culture. Our previous studies indicated that enterally feeding *L. reuteri* is associated with recovery of the plasma levels of inosine and hypoxanthine to levels similar to WT, at the same level that inosine levels decreased in the stool of these mice (7). We hypothesized that, most likely, *L. reuteri* promotes inosine absorption in the intestine by improving overall gut health through multiple mechanisms (for example, by improving villus length) and/or by modulating the gut microbial community. We measured the small intestinal villi in SF mice compare with SF mice after oral feeding *L. reuteri* and showed that orally feeding *L. reuteri* improves the length of villi and depth of crypts. Furthermore, an increased expression of equilibrative nucleoside transporter transporters after *L. reuteri* feeding was found, which could contribute to produce improved absorption. The best method to confirm enhanced absorption would be to orally feed labeled inosine after administration of *L. reuteri* and quantify the labeled inosine in the circulation. However, the labeling approach for small molecules like inosine is much more difficult than for amino acid or proteins. In the meantime, we could not rule out that *in vivo* the gut environment could activate the enzymes such as adenosine deaminase (ADA) and 5′-nucleotidase generated by *L. reuteri* to produce inosine. But it is difficult to distinguish the ADA activity in the intestinal tissue lysates from the activity of *L. reuteri* or other microbes, because ADA activity is very high in the intestine (44). The direct links between *L. reuteri* and the metabolites required further exploration.

We also noticed that A_2A_ receptor appears to be expressed in other organs besides lymphocytes (11, 45). In liver, the A_2A_ receptor is expressed in Kupffer cells, hepatocytes, and hepatic stellate cells (46–48). Some studies suggested that the A_2A_ receptor plays a role not only in regulating inflammation but also in maintaining liver function in general (39). Previous studies also revealed that it is more highly expressed in spleen, lymph nodes, liver, and lung than that in the small intestine or adrenal gland, supporting a functional role of this receptor in the regulation of the immune response in peripheral lymphoid tissues (11). It has been reported that A_2A_ receptor activation confers tissue protection in peripheral organs (49, 50). While the mechanism of *L. reuteri* in regulating inflammation in SF mice clearly involves T cell modulation, we cannot rule out that A_2A_ receptor expression in these organs may also contribute to the beneficial effects of *L. reuteri* in SF mice. Therefore, A_2A_ receptor expression on both immune cells and other cells and their interaction may determine the overall impact of A_2A_ receptor deletion on beneficial effects of *L. reuteri*.

The role of the T cell and its expression of A_2A_ modulated by *L. reuteri* or highly related metabolites such as inosine could be further studied by using a T cell knockout mouse model by adoptive transfer of CD4^+^ T cells isolated from WT, SF, ${\text{A}}_{{\text{2A}}^{\text{-/-}}}$, or SF$\cdot {\text{A}}_{{\text{2A}}^{\text{-/-}}}$ with/without *L. reuteri* or inosine treatment, which is currently under investigation.

In summary, our study demonstrates that adenosine A_2A_ receptor deletion does not inhibit the development of autoimmune disease in the SF mouse. However, adenosine A_2A_ receptor deletion reverses the inhibition of *L. reuteri* on autoimmunity induced by Treg-deficiency in SF mice. Our results support the concept that activated adenosine A_2A_ receptors are linked to *L. reuteri* effects *in vivo*. They also suggest that the activated A_2A_ receptor by *L. reuteri* or other agonists may represent a useful therapeutic strategy for preventing lethal outcomes in Foxp3 deficency- or dysfunction-induced autoimmune diseases.

## Ethics Statement

This study was carried out in accordance with the recommendations of the Guide for the Care and Use of Laboratory Animals (NIH) and The Institutional Animal Care and Use Committee (IACUC). The protocol was approved by the IACUC (Protocol number: AWC-14-056 and AWC-17-0045).

## Author Contributions

BH, YL, and JR conceived and designed the experiments. BH, TH, and YL performed all experiments and analyzed the data. BH, YL, DT, and JR wrote the paper and edited the manuscript. All authors read and approved the final manuscript.

## Conflict of Interest Statement

The authors declare that the research was conducted in the absence of any commercial or financial relationships that could be construed as a potential conflict of interest.
