# Supplementary figures and images for: Adenosine A2A Receptor Deletion Blocks the Beneficial Effects of Lactobacillus reuteri in Regulatory T-Deficient Scurfy Mice
Source: Front Immunol. 2017 Dec 6;8:1680. doi: 10.3389/fimmu.2017.01680 (PMC5723640; doi:10.3389/fimmu.2017.01680)

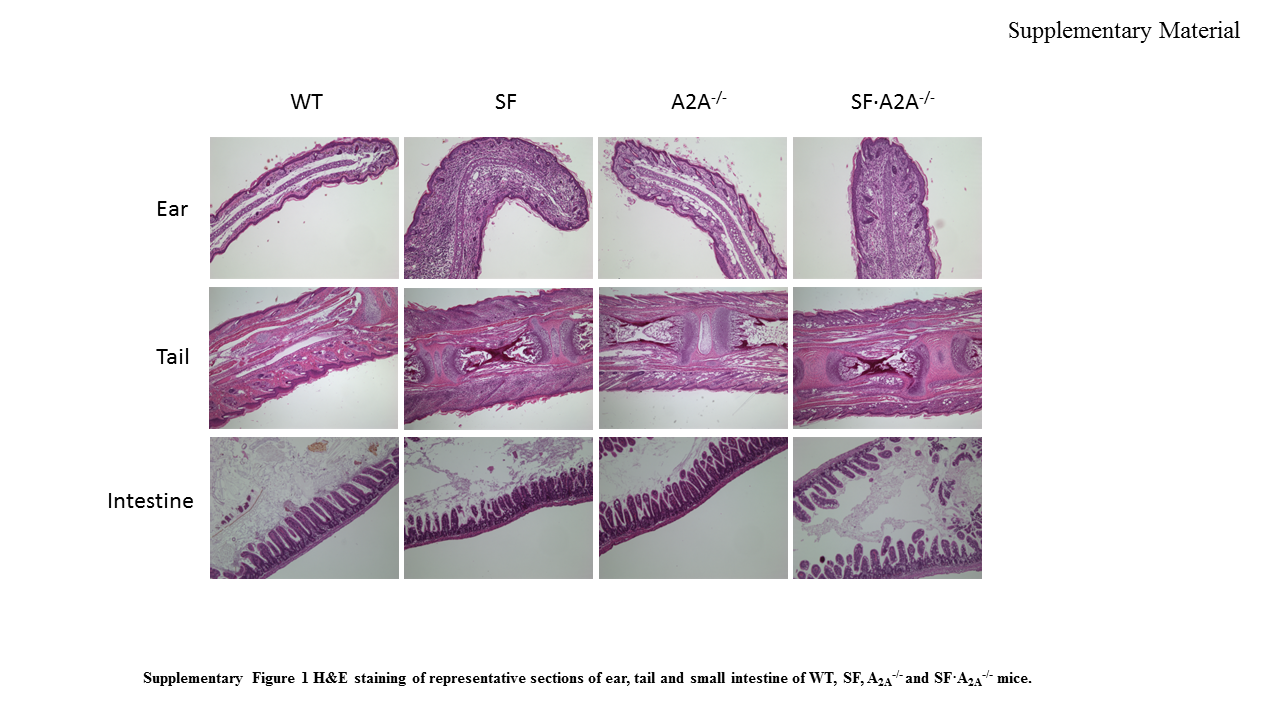

Supplement: Supplementary file 1 [file Image_1.tif]

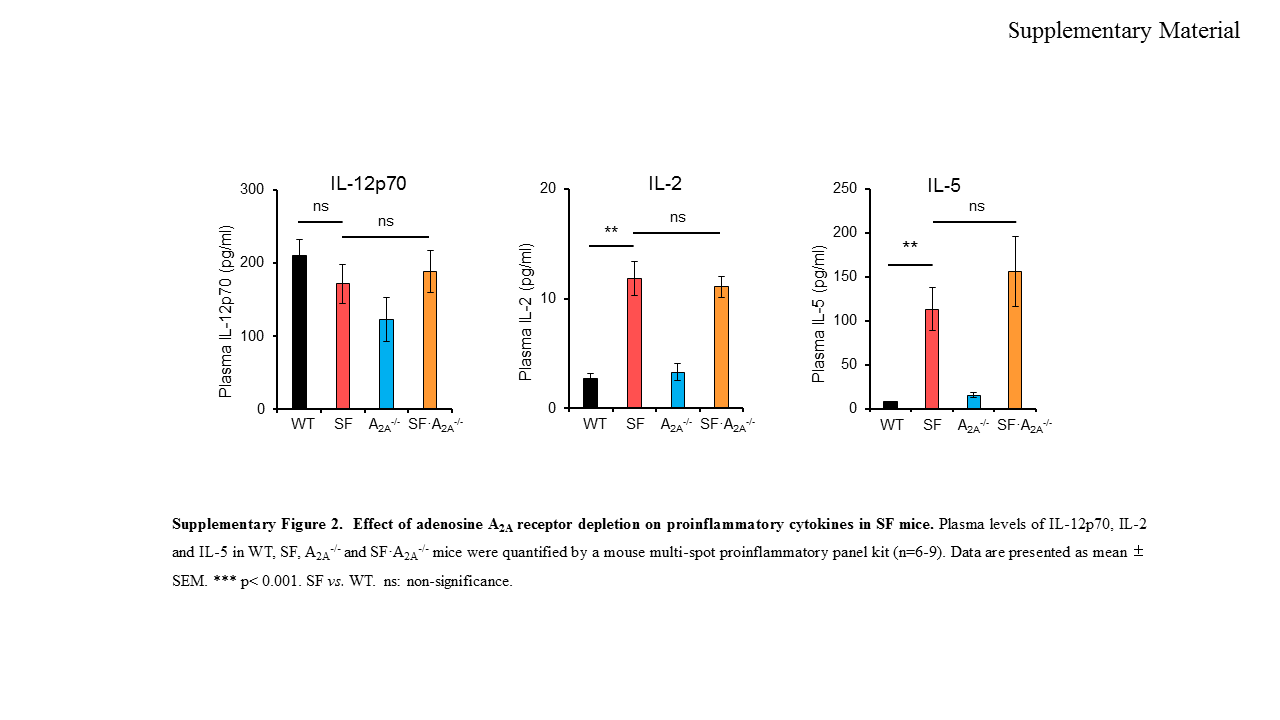

Supplement: Supplementary file 2 [file Image_2.tif]

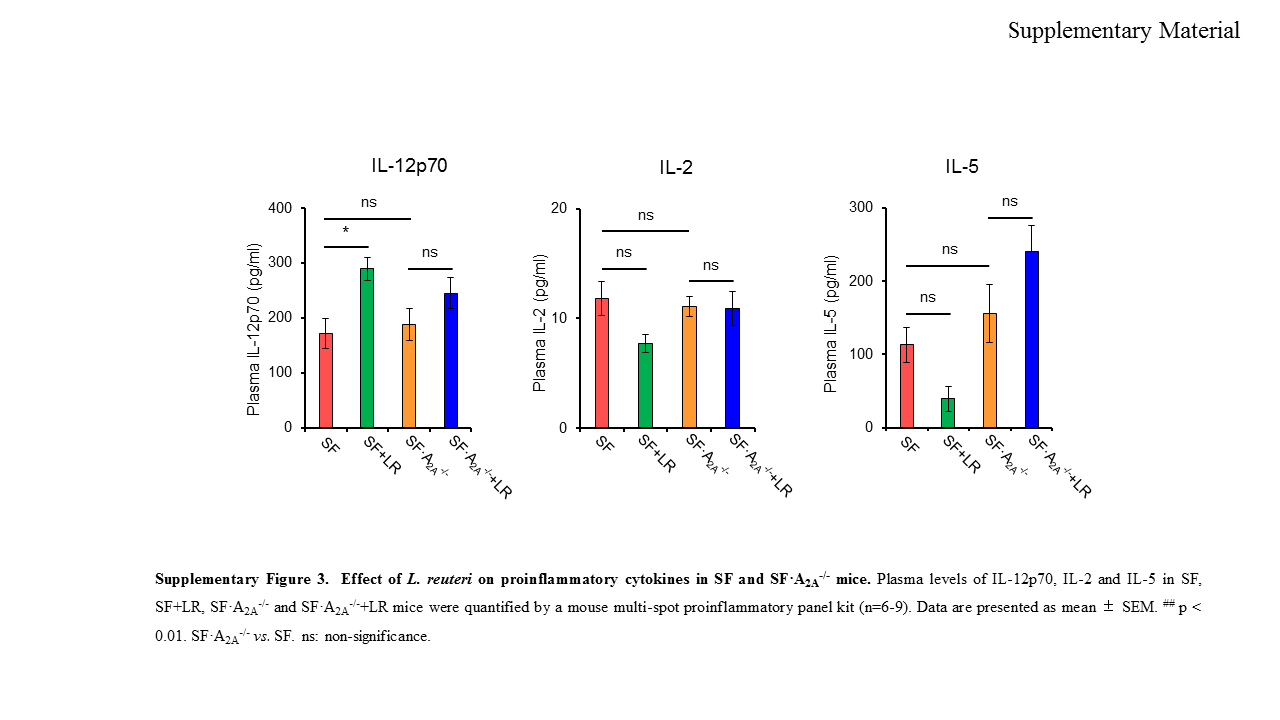

Supplement: Supplementary file 3 [file Image_3.tif]
